# Supplementary figures and images for: Perturbations in gut microbiota composition in schizophrenia
Source: PLoS One. 2024 Jul 3;19(7):e0306582. doi: 10.1371/journal.pone.0306582 (PMC11221673; doi:10.1371/journal.pone.0306582)

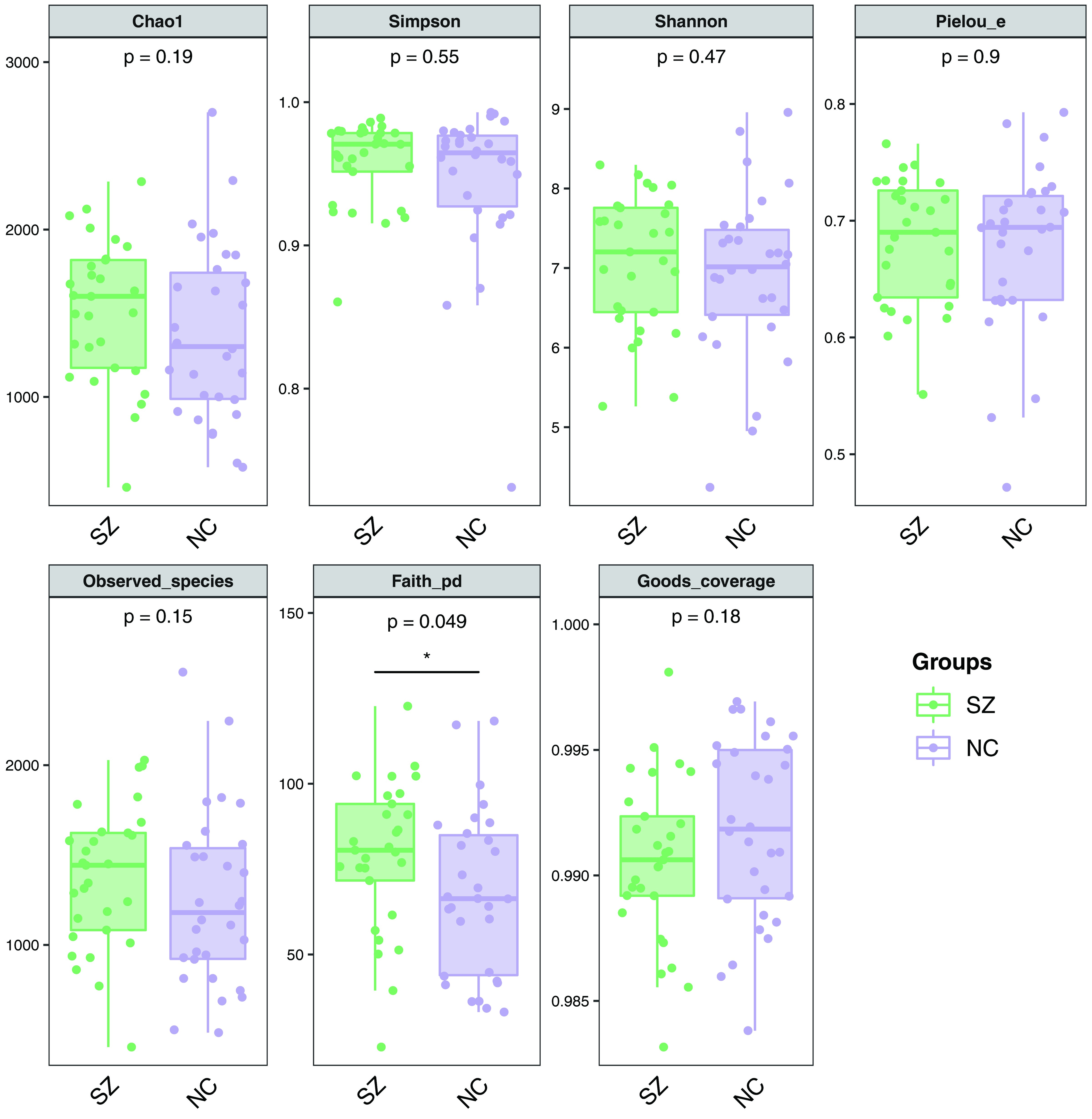

Supplement: S1 Fig — The alpha diversity between two groups, including Chao1, Simpson, Shannon, Pielou_e, Observed_species, Faith_pd, and Goods_coverage. Note: SZ: Schizophrenia; NC: Normal Controls. (TIF) [file pone.0306582.s001.tif]

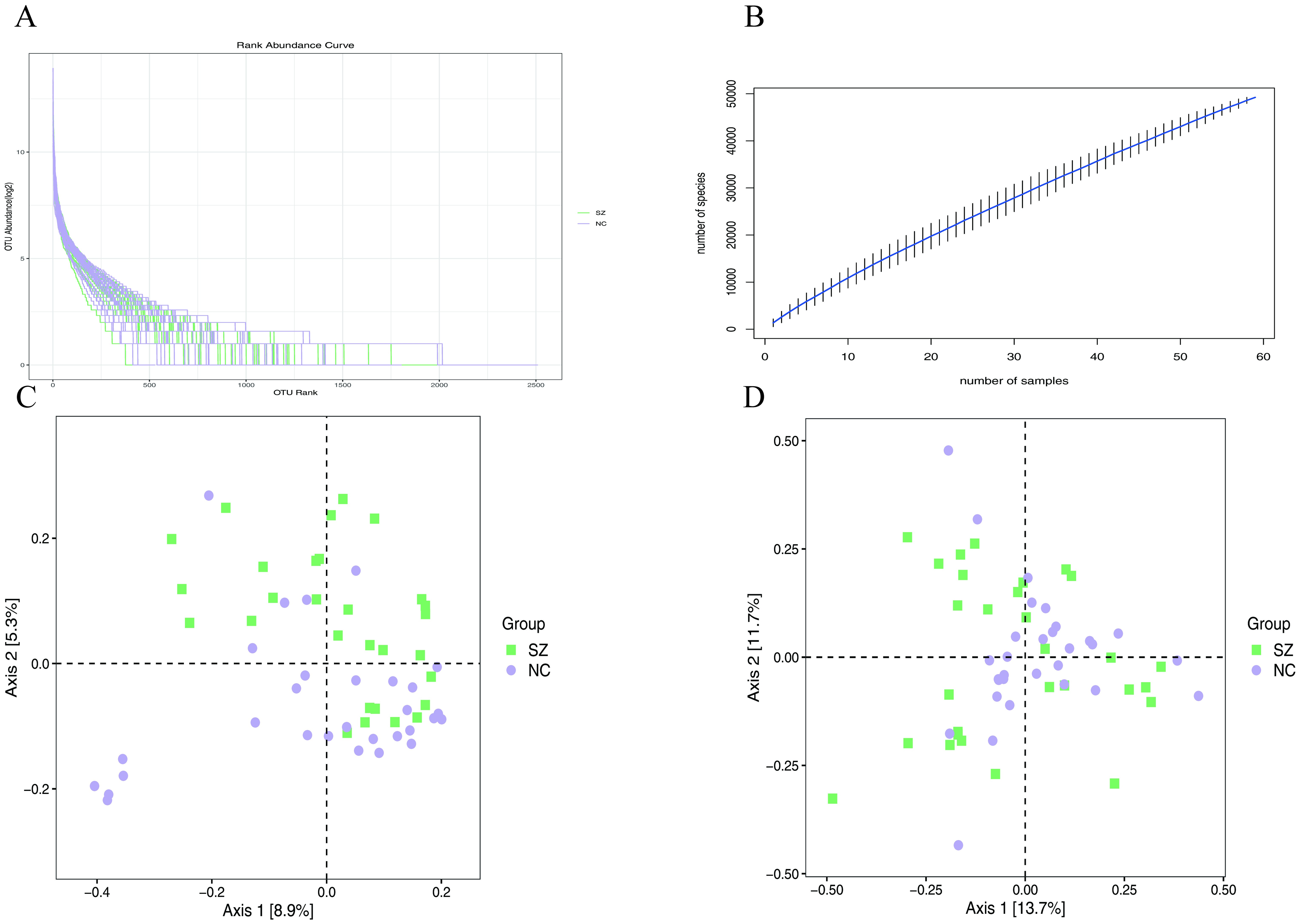

Supplement: S2 Fig — (A) Rank abundance curve. (B) Species accumulation curves. The β-diversities of SCZ and NC. (C, D). PCoA of Unweighted and weighted Unifrac Distance at the OTU level. Note: SZ: Schizophrenia; NC: Normal Controls. (TIF) [file pone.0306582.s002.tif]

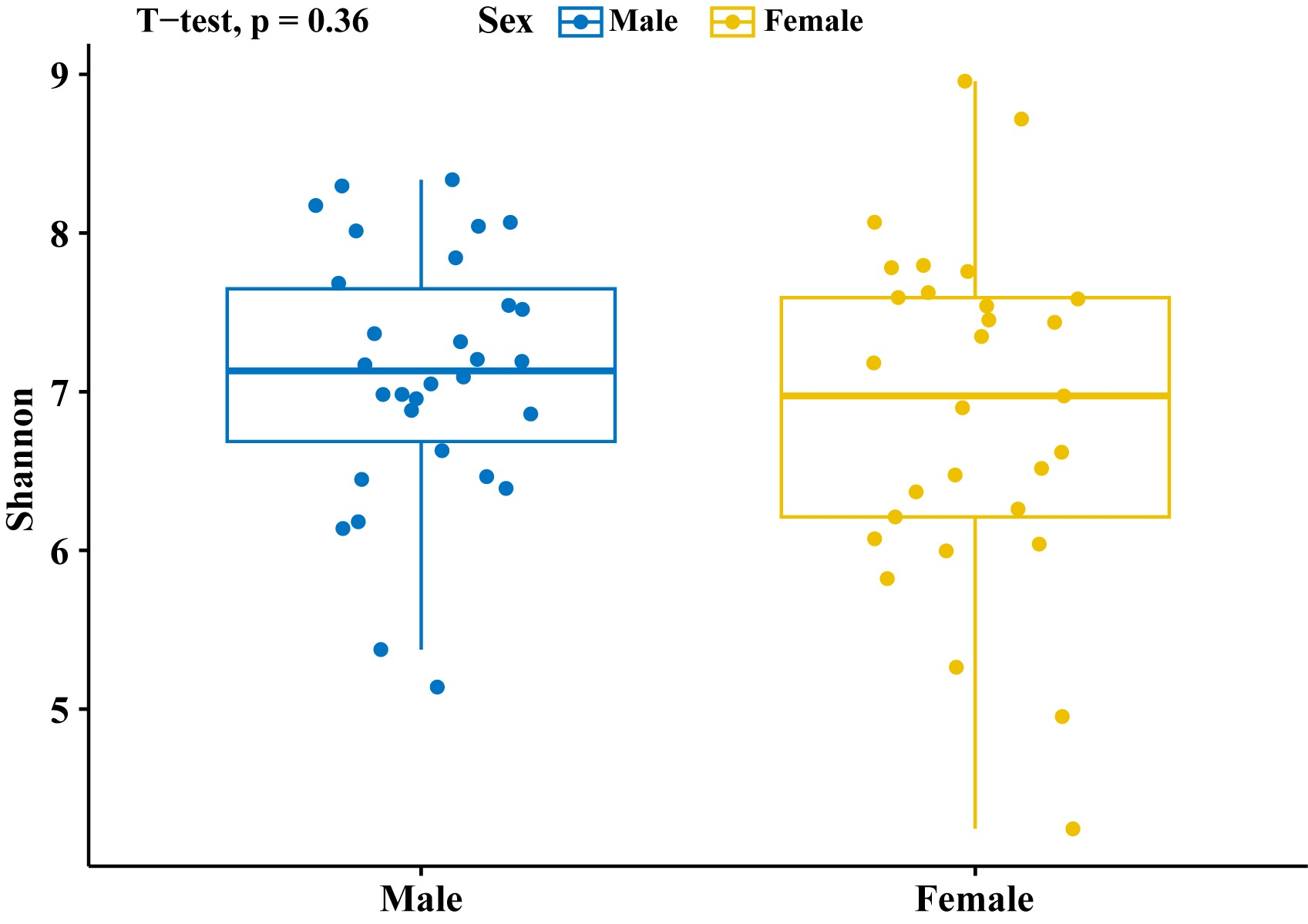

Supplement: S3 Fig — (TIF) [file pone.0306582.s003.tif]
