# Supplementary material for: Perturbations in gut microbiota composition in schizophrenia
Source: PLoS One. 2024 Jul 3;19(7):e0306582. doi: 10.1371/journal.pone.0306582 (PMC11221673; doi:10.1371/journal.pone.0306582)
Supplement: Attachment — Submitted filename: Response to reviewers.docx [file pone.0306582.s004.docx]

**Dear editor:**

We feel great thanks for your professional review work on our article. As you are concerned, there are several problems that need to be addressed. According to your nice suggestions, we have made extensive corrections to our previous draft, the detailed corrections are listed below.

**Reviewer #1:**

**Q1**. The authors have mentioned in the abstract that “The results of the functional analysis revealed a significant correlation between the metabolism of sphingolipid, phosphonates, and phosphinates, as well as glutamine metabolism, and the manifestation and progression of schizophrenia”. However, these results need to be elaborated in the results section. Moreover, the authors must discuss the effect of phosphonates and phosphinates pathways as well. Although some potential pathways are identified, the functional mechanisms linking specific microbes to schizophrenia remain unclear.

**A:** According to your nice suggestions, we have made extensive corrections to our incorrect conclusions in the abstract section.

**Q2**. The study lacks a longitudinal design, which could provide insights into the dynamic changes in the gut microbiome over time in schizophrenia patients. Moreover, very sample size especially for a metagenomics study makes statistical power and generalizability weak and unreliable.

**A:** We agree that more studies would be useful to understand the relationships between the microbiota with the onset and progression of schizophrenia. Due to the heterogeneity of individual differences and treatment methods, no longitudinal design was conducted for enrolled cases. In the subsequent experiments, we will expand the sample cohort to provide more comprehensive data support.

**Q3.** The exclusion criteria for patient selection seem comprehensive but could benefit from further elaboration on how these criteria were applied and any potential biases introduced.

**A:** We sincerely thank the reviewer for careful reading. In order to increase the homogeneity of the study population and to reduce the risk of adverse events and bias arising from competing risks, exclusion criteria were developed to allow screening based on patients' general data. Appropriate exclusion criteria, rather than strict exclusion criteria, would enable us to obtain a sufficient number of cases for the study and more specific exclusion criteria may require more clinical evidence. We will continuously follow the latest research findings in this field and improve the inclusion and exclusion criteria for patients in subsequent studies.

**Q4.** The methodology section should be more detailed, particularly regarding the data analysis pipelines for both 16S rRNA sequencing and metagenomic analysis. What are the chances of having human DNA contamination when extracted genomic DNA from fecal samples and how it was avoided?

**A:** More detailed illustrations for both 16S rRNA sequencing and metagenomic analysis has been added in the methodology section. About phase “human DNA contamination” is a writing mistake and has been revised as follows: “deplete host DNA”.

**Q5.** The authors have limited discussion on Selenomonas. This bacterium shows significant enrichment in the schizophrenia group, therefore comparison with existing literature is needed.

**A:** The description of Selenomonas have been removed from the results and discussion section.

**Q6.** In lines 432-433, the authors mentioned “This investigation demonstrates that patients with schizophrenia exhibit abnormal glutamate metabolism”, however, abnormal glutamate metabolism is not mentioned in the results section. Elaborate on this point in the results and then mention it in the discussion otherwise, it is a very confusing statement.

**A:** The illustration of “glutamate metabolism” has been added both in the results and discussion section.

**Q7.** As the patients were of mixed gender, please state if there is any gender-specific difference in microbiota and if is related to some specific pathway.

**A:** The statistic analysis of gender-specific difference in microbiota has been added in the results section on line 266-267.

**Q8.** Line 449-450: Please explain how your findings provide a potential mechanism explanation for the observed sequencing data while no mechanisms have been explained.

**A:** The mechanisms explanation has been added in the discussion section on line 421-447.

**Q9.** Add high-quality pictures because the text in the Figures is not readable in the current format**.**

**A:** The figures has been revised.

**Q10.** In lines 355-358, the aim of the study does not match the aim mentioned in the abstract and the objective mentioned in lines 118-119. The statement could be modified by saying that “one of the aims of this study was to investigate…..”

**A:** According to your suggestion, we have unified this statement.

**Q11.** The authors have focused more on microbiota comparison. Overall, metagenomics analysis needs to be elaborated and correlated with pathways of the microbiome that respond to SZ in more detail to present clear results of your findings.

**A:** The illustration about the pathway has been added in the discussion section.

**Q12.** The article demonstrates proficiency in English language usage, with coherent sentences and technical terminology. However, there are some minor grammar issues and typographical errors that could be corrected for clarity and precision. For example, line 300: "worthing" should be "worth mentioning," and "differentially abundance" should be "differentially abundant." Line 69: “exerts a significant burden on the global population” could be “exerts a significant burden on the global health sector”. Line 210: delete one “control”; line 237: revealed or showed, delete one word; lines 428-429: Previous research??

**A:** We tried our best to improve the manuscript and made some changes to the manuscript.

**Q13.** All references are not in the same format. There are no details for reference numbers 6 and 7.

**A:** We have revised the reference. Reference 6 is a index of the book.

**Major concerns:**

**Q1.** In introduction, the authors did not cite any original studies and review articles on the microbiota study of schizophrenia, which is misleading. The author should present and give credit to previous pertinent work, before highlighting what their data adds to the existing knowledge.

**A:** We sincerely appreciate the valuable comments. We have checked the literature carefully and added reference on 12-14 into the introduction part in the revised manuscript.

**Q:2.** The sequencing reads number of each sample in 16s rRNA gene sequencing should be presented. And was the data normalized before analysis? Since you identified 30 unique genera in patients or control (Line 238-245), the sequencing depth must be enough to support your findings.

**A:** The information of sequencing reads has been added in the results section on line 242-250.

**Q:3.** In Line 262-269, the authors compared the two groups using LEfSe. Then in Line 270-276, the authors used Wilcoxon rank-sum test to compare them again. It’s un-necessary. Same problem existed in metagenomic analysis. I suggest the authors use multiple wilcoxon ranked sign tests to compare the microbial abundances between the groups with calculation of FDR.

**A:** We have rewritten this part in the results section.

**Q: 4.** There are logical issues with metagenomic analysis. I don’t think 16s rRNA gene analysis result should be validated by metagenomic analysis. And the authors did not compare the taxa profiles obtained by this two different methods.

**A:** We are sorry for our logical issues, we have made the corrections to these false claims within the whole manuscript.

**Q:5.** The results should be separated into several parts to make it clear and readable.

**A:** In the results section, we added sub-headings to make the article easier to read.

**Q:6.** The title must be changed. Indeed the authors only studied gut microbiota, which can not link lipid metabolism and cognitive disorder.

**A:** Thank you for the title suggested. The precedent version of the title has been replaced, becoming **perturbations in gut microbiota composition in schizophrenia.**

**Minor concerns:**

**Abstract**

**Q:1.** line31, ‘the of’ is wrong

**A:** We were really sorry for our careless mistakes. Thank you for your reminder.

**Q:2.** Change ‘16S rRNA’ to ‘16S rRNA gene’ throughout the manuscript.

**A:** Thanks for your careful checks. We are sorry for our carelessness. Based on your comments, we have made the corrections within the whole manuscript.

**Introduction**

**Q:3.** Please separate the 1ST paragraph into different parts.

**A:** Thank you for pointing this out. The 1 ST paragraph has been separated into different parts based on the statement content of each paragraph.

**Q:4**. Microflora should be no longer used in the literature, please change it into microbiota.

**A:** Thank you for pointing this out. The microflora has been corrected on microbiota.

**Methods**

**Q:5.** Line 186, 2 x250?

**A:** We were really sorry for our careless mistakes. Thank you for your reminder.

**Q:6** Line 193, details of the 12 participants should be given, such as xx controls and xx patients

**A:** Thank you for pointing this out. The reviewer is correct, and we have revised it as follows “we selected a cohort of 12 participants **including 6 patients with schizophrenia and 6 healthy controls”.**

**Q:7.** The analysis method of 16s rRNA gene sequencing is absent.

**A:** It is really a giant mistake to the whole quality of our article. We feel sorry for our carelessness. We have corrected it and added this section in the manuscript.

**Q:8.** More details about metagenomic analysis, such as pathway prediction, should be presented.

**A:** Thank you for pointing this out. More details about metagenomic analysis have been added in the manuscript.

**Q:9** The raw sequencing data should be deposited in a publicly available database.

**A:** Thank you for your suggestions. **All raw sequences were deposited in NCBI Sequence Read Archive under accession number PRJNA 1077638 for metagenomics sequencing and PRJNA 1077648 for 16S rRNA gene sequencing.**

**Results**

**Q:10.** Line 209-210, is this a new cohort, or samples selected from the 59 volunteers?

**A:** More details about the participants enrolled in metagenomic sequencing section were added in the manuscript. The revised text reads as follows: We selected a cohort of 12 participants **including 6 patients with schizophrenia and 6 healthy controls from 59 enrolled samples** whose feces samples provided sufficient material for a metagenomic analysis.

**Q:** **11.** Line 237, ‘revealed showed’?

**A:** We are really for our careless mistakes. The duplicate verbs have been removed.

**Q:12.** Line 249, ’30 genera’ is not correct. It’s 198 in Figure 2A.

**A:** As for the identification of strains, we have recognized and modified the whole process.

**Q:** **13. Line 251, right parenthesis is absent.**

**A:** We are really for our careless mistakes. We have made changes to the sentences as follows: *selenomonas, mogibacterium,* and *corynebacterium* were found to be dominant **with each genus comprising more than 1% of the total microbiome**.

**Q:** **14.** Line 262-265, move into method part.

**A:** Thank you for your suggestions. The section of “LefSe analysis” has been moved from the results section to the methods section.

**Q:15.** Line 291, ‘Suoliviridae’ is not a genus name but family.

**A:** We are really for our careless mistakes. We have made changes to the sentences as follows: family *Suoliviridae*.

**Q:16.** Line 300-302, it’s not suitable in Results, please delete it or move into Discussion.

**A:** We have moved this section moved into Discussion.

**Q:** **17.** Line 302-305, ‘Clostridium may exert a significant influence on the etiology and progression of schizophrenia’ is not supported by your data.

**A:** We have made more appropriate changes to the sentence as follows: suggesting that Clostridium may play an important role in the progression of schizophrenia.

**Q: 18.** Line 319-324, PICRUSt is based on 16s rRNA gene prediction. But you should also present your pathway analysis based on metagenomics, which would provide strong support to this prediction.

**A:** As suggested by the reviewer, we have presented our pathway analysis based on metagenomics in the results section.

**Q:** **19.** Figure 1, change ‘16s rDNA gene’ into ‘16s rRNA gene’. Line 225, change ‘16S rDNA’ into ‘16s rRNA gene sequencing’. Line 226, ‘Clean reads without human sequences were blasted to NR databases.’ seems weird, please re-write this part.

**A:** We feel sorry for our carelessness. We have re-written this part as follows: Clean reads were blasted to NR databases.

**Q: 20.** Table 2, it’s genus, not OTU.

**A:** We feel sorry for our carelessness. We have corrected the “OUT” into “genus”.

**Discussion**

Line 347-355, what you stated is not the truth. Please revise this part.

A: We have revised the discussion section throughout.
