# Supplementary material for: Perturbations in gut microbiota composition in schizophrenia
Source: PLoS One. 2024 Jul 3;19(7):e0306582. doi: 10.1371/journal.pone.0306582 (PMC11221673; doi:10.1371/journal.pone.0306582)
Supplement: Attachment — Submitted filename: Response to Reviewers.docx [file pone.0306582.s005.docx]

**Response to Reviewers:**

**Reviewer 3**

**Q1:** L 41-42: Based on your results, we can only conclude a relationship between gut microbiota with schizophrenia, rather than a crucial role.

**A:** We have modified this overly positive sentence.

**Q2：**L 101: collected what? This is an incomplete sentence.

**A:** According to the reviewer’s comment, we have corrected the sentence.

**Q3:** L 216: why you only analysis the function of metagenomics, the diversity and composition of microbial community, even the resistome can also be obtained.

**A:** We are sorry for our carelessness. The section of analysis of the 16S rRNA gene sequencing has been added in the text.

**figures: the resolution of figures was low.**

**A:** Fig 1 has been redrawn, and all figures has been upload to the PACE digital diagnostic tool to meet PLOS requirements.

**Reviewer 4**

**Q:1** In abstract, the authors summarized that “These findings suggest that gut microbiome may exert a crucial influence on individuals with schizophrenia” . I think more important results should be described to support this conclusion, not just “minimal variations in alpha and beta diversity” or “higher abundances of Clostridium and Megasphaera”.

**A:** We have made changes to the abstract section.

**Q:2** In introduction, authors simply stated “Several studies have investigated the microbiota in schizophrenia, revealed its relationships to the onset and progression of schizophrenia”. The detailed findings of previous study are supposed to be listed.

**A:** Thank you for your suggestions. The detailed findings of previous study has been added in the manuscript.

**Q:3** In the exclusion criteria, schizophrenia patients with chronic gastrointestinal illness were not ruled out, which might affect the study results.

**A:** A reference to chronic gastrointestinal illness has been added to the exclusion rule.

**Q:4** Figures in the manuscript were fuzzy. Please provide clearer figures.

**A:** All figures have been upload to the PACE digital diagnostic tool to meet PLOS requirements.

**Q:5** In fig 2A, authors compared the microbial compositions between group SZ and group NC, and in abstract, authors found that “The schizophrenia patients showed higher abundances of Clostridium and Megasphaera” , however, whether the data were statistically significant were not mentioned.

**A:** Statistical analysis and comparison of *Clostridium* and *Megasphaera* were shown in **Fig 3F**.

**Q:6** In discussion, authors didn’t highlight the important findings of the study, analyze the reasons for observed data or compare the research findings with previous studies.

**A:** The findings has been added in the discussion section, and the comparison of the research with previous studies were illustrated on references 26-29.

**Q:7** Limitations were not mentioned in the discussion.

**A:** Limitations of the study were added in the conclusion section of the manuscript.

**Q: 8** The current manuscript needs to be polished by a native English speaker or a professional language editing service.

**A:** Thanks for your suggestion. We have tried our best to polish the language in the revised manuscript. The article has been sent to textcheck for polishing, and the certificate can be seen at http://textcheck.com/certificate/index/KihIW2.
